# Cervical precancerous lesions at the Tchibanga Regional Hospital and the University Hospital in Gabon in 2018: smartphone as a screening tool for diagnosis

**DOI:** 10.4314/ahs.v22i1.12

**Published:** 2022-03

**Authors:** Nathalie Ambounda Ledaga, Sylvain Honore Woromogo, Felicite Emma Yagata-Moussa, Audin Serge Mavoungou, Vicky Noel Simo Tekem

**Affiliations:** 1 Inter State Centre for Higher Public Health Education in Central Africa (CIESPAC), Brazzaville, Congo; 2 University Hospital Centre of Libreville, Gynecology and Obstetric Service, Gabon; 3 Faculty of Health Sciences, University of Bangui, Central African Republic

**Keywords:** Diagnosis, cervical precancerous lesions, Tchibanga, smartphone

## Abstract

**Background:**

Cervical precancerous lesions are disorders that can induce discolouration changes. Their detection is difficult in remote areas in the absence of adequate equipment. The objectives were to evaluate Smartphone performance in diagnosing cervical precancerous lesions in Tchibanga, Gabon.

**Methods:**

It was an interventional cross-sectional study to evaluate the validity and reliability of the smartphone as a tool for diagnosing atypical changes in the cervix. Study period was between July 1, 2017 to February 28, 2018 at the Tchibanga Regional Hospital (CHRT) and the University Hospital (CHU). The variability between examiners was determined according to Cohen's Kappa formula. The Gold standard test was the cytology.

**Results:**

Compared to the examiner -1, the examiner - 2 found a high percentage of inflammations as atypical transformations : 15.3% versus 9%. With regard to smartphonic impressions, the examiner-1 found the normal impressions almost equal to that of the examiner-2, 72.9% versus 72.2%. The concordance between positive smartphonic impressions was 93.8% and 95.5% between negative smartphonic impressions, with k = 0.86.

**Conclusion:**

In view of the above, the concordance between positive and negative smart phonic impressions was 93.8 and 95.5% with k = 0.86. The performance parameters being good, there is a need to use the smartphone as a tool for the diagnosis of precancerous lesions.

## Introduction

Cervical precancerous lesions Precancerous cervical lesions are changes to the cervical cells in an area called the transformation zone. According to the World Health Organization (WHO), this change can exist at any of three stages: cervical intraepithelial neoplasia stage 1 (CIN1), stage 2 (CIN2), or stage 3 (CIN3). These conditions are not yet cancer but improving screening coverage, close management and follow-up could which decrease the morbidity and mortality caused by cervical cancer^1^–^3^. Visual inspection after application of acetic acid and lugol is facilitated by colposcopy and in most cases, grade 2 AT reflect the presence of precancerous lesions^4^. Globocan estimates that in 2018 there was approximately 570,000 new patients and more than 311,000,000 deaths of women each year from cervical cancer worldwide. More than 85% of these deaths occur in low5 or middle-income countries where the prevalence of precancerous lesions is 2.5% 6.

In Gabon, cervical cancer represents 26.3% of female cancers; early detection of this disease through screening has been implemented in primary and secondary health facilities in 9 of 10 regions of the country since 2014 ^7,8^. Despite the increase in the country's computer development index, Nyanga province is the only one that does not benefit from cervical cancer screening. To this end, the Smartphone has been used as a tool for the diagnosis of precancerous lesions of cervical cancer. Few data are available on the evaluation of the performance of the Smartphone as a screening tool in the diagnosis of atypical cervical transformations in Tchibanga, Gabon. The objective of this study is to evaluate Smartphone performance in diagnosing precancerous cervical lesions in Tchibanga, Gabon in 2018.

## Objective

Evaluate Smartphone performance in diagnosing cervical precancerous lesions in Tchibanga, Gabon in 2018.

## Methods

### Study design

This was an interventionally oriented cross-sectional study that assessed the validity and reliability of the smartphone as a tool for diagnosing atypical cervical transformations. It took place from July 1, 2017 to February 28, 2018, i. e. eight months, at the Regional Hospital Benjamin Ngoubou of Tchibanga (CHRBNT) and the University Hospital (CHU), which housed the pilot centre for the early detection of breast and cervical cancer in Libreville. The CHRBNT did not have a cervical cancer screening unit.

### Study population

The study population consisted of sexually active women who signed the informed consent form after the cervical cancer awareness campaign. To determine the sample size of this study, we used Daniel SCHWARTZ's formula9. We considered the prevalence (P) of precancerous lesions in Guinea Conakry as 2.6%5 because of the similarity in research methodology; the alpha risk (α) as 5% and the precision (i) as 5%. N = p (1-p) zα^2^ /i^2^, we found 144 participants. The data were collected using a questionnaire.

### Inclusion criteria

In this study, women volunteers who had signed the informed consent form were included.

### Exclusion criteria

Women with a history of complete hysterectomy, follow-up cervical cancer, and those who were pregnant or postpartum were excluded from this study, as were refusals.

### Sampling procedure

An awareness and information campaign on cervical cancer was conducted in the commune of Tchibanga, particularly in churches, mosques and at the CHRBNT. Women were recruited on a first-come, first-served basis during the study period.

We have received authorization from the Ministry of Health and Population of Gabon to conduct this campaign.

### Diagnostic method and material

The diagnosis was conducted by two examiners. At the Tchibanga site, it was performed by a 7^th^ year medical student trained in the technique of visual inspections of the cervix and colposcopy for six weeks. The latter was supervised by a gynaecologist-obstetrician and a general practitioner. At the University Hospital Centre in Libreville, the diagnosis was made by a gynaecology-obstetrician trained in colposcopy by the World Health Organization, LAlla SALMA Foundation and the International Agency for Research on Cancer (IARC) in Barshi, India and Egypt.

The usual standard screening equipment was used, in addition to a smartphone. The technical equipment for screening included an examination room with a register, a good quality light source, a gynaecological examination table, a step stool, a folding screen, consumables for VIL/VIA and for cervical cytology. The Smartphone was powered by an LED photo/video camera, with a resolution of 13 megapixels, 16 million colours manual with autofocus settings. An interview specified the patient's socio-demographic profiles and gynecoobstetrical and medical antecedents, recorded on a patient record. Then a speculum examination was carried out during which successively an unprepared visual inspection, a visual inspection based on acetic acid (VIA) and a visual inspection based on Lugol (VIL) were performed.

A photograph enlarged by median zoom (×2) was taken in all three times, with the smartphone placed about 15 cm from the cervix. The images were then transferred to a computer, coded on the last section of the data collection sheet for the smartphone exam and sent by e-mail to the various examiners. The results between the two examiners were obtained blindly. This form contained only the code number, age and hormonal status of the woman as information. When an anomaly was found on VIA/VIL images, the woman was seen again after 48 hours for a uterine cervical smear test performed by a trained senior medical biology technician. Image quality criteria were defined by the sharpness, brightness and visibility of the anatomical elements of the cervix (Pavimento Cylindrical Junction; External Cervical Orifice; endocervix and ectocervix).

### Data analysis

The data were analyzed using the Statistics Package for Social Sciences (SPSS) version 20 software. Absolute and relative frequencies as well as central trend parameters (mean, median) and dispersions were calculated. The variability between examiners was determined according to Cohen's Kappa formula (*k = Pa - Pc* / 1 - *Pa*) where *Pa* is the observed proportion of agreements, *Pe* the theoretical proportion of agreements observed. For the purpose of clarity, the term “smart phonic impression” was used in this study to refer to the appearance of the cervix on a smartphone image. Regarding the performance of the test, we used the cytology as Golden test. The performance (Sensitivity, specificity, positive and negative predictive value (PPV, NPV)) of the Tchibanga field tests were calculated as well as their 95% confidence interval.

### Ethical considerations

We received authorization from the Ministry of Health and Population of Gabon to conduct this campaign. The study was conducted in accordance with the Good Clinical Practice (GCP) guide and the regulations of the Ministry of Public Health and Population. Screening by visual methods is authorised by the Gabonese health authorities and institutions, according to WHO recommendations. Women gave written consent before their cervix was photographed. The images were coded before transfer and remained strictly anonymous.

## Results

### Socio-demographic, reproductive and clinical characteristics

A total of 144 women participated in the study, the median age was 34 years with extremes from 18 to 70 years. Married women accounted for 77.8% (n=112), of whom 7.6% (n=11) came from households with a family history of cervical cancer (Table 1).

**Table 1:** Socio-demographics and clinical characteristics of participants

Characteristics	Participants N=144	
	
	n	%
Age		
Range age (years)	(18 ;70)	
Average age	34 years ± 3.2	
Median age (Q1 ; Q3)	34 years (23 ; 61)	
18–24	46	32.0
25–34	36	25.0
35–44	27	18.8
45–54	28	19.4
55–70	07	4.8
Marital status		
Married	112	77.8
Unmarried	26	18.0
Widows	06	4.2
Hormonal status		
Menopausal women	19	13.2
Premenopausal	125	86.8
Family antecedents		
Uterine cancer	11	7.6
Breast cancer	06	4.3
Others *	07	4.8
No cancer	120	83.3
Parity		
0	49	34.03
1	34	23.61
2–3	16	11.11
> 3	45	31.25
Histiry of STIs		
Chlamydia	57	39.58
HIV	07	4.86
Others	19	13.20
None /unknown	61	42.36

* Leukaemia, oropharyngeal cancer

**Table 2:** Characteristics of the smartphonic images analysed on site 1 and 2

Characteristics	Examiner 1(on site) N=114			Examiner 2 (off site) N=114
	
	n	%	n	%
Lesions types				
Inflammation (red)	13	9.0	22	15.3
Others	02	1.4	02	1.4
No lesion	129	89.6	120	83.3
Connecting area				
Visible	136	94.4	134	93.0
Partially visible	02	1.4	04	2.8
Not visible	06	4.2	06	4.2
Images after VIA				
Positive	19	13.2	21	14.6
Negative	125	86.8	123	85.4
VIL image				
Positive	35	24.3	32	22.22
Negative	109	75.7	112	77.78
Smart phonic impressions				
ATG1	24	16.7	23	16.0
ATG2	11	7.6	09	6.2
Normal impressions	105	72.9	104	72.2
Others (cervical inflamation)	04	2.8	08	5.6

ATG: Atypical Transformation of Grade

**Table 3:** Concordance between the two sites regarding VIA, VIL and smartphonic impressions results

Examiner1(on site)	Examiner 2(off site)			
			
	Positive test	Negative test	Total	k
Acetic acid inspection (VIA)				0.83
VIA test positive	17	2	19	
VIA test negative	4	121	125	
Total	21	123	144	
Lugol-based inspection (VIL)				0.86
VIL test positive	30	05	35	
VIL test negative	02	107	109	
Total	32	112	144	
Smartphonic impression				0.86
Impression+	30	05	35	
Impression-	02	107	109	
Total	32	112	144	

**Table 4:** Performance of VIA/VIL tests at the Tchibanga site in 2018 compared to cytology results

Test to explore	Reference test (cytology)				Total N=135	Sensitivity (95 % CI)	Specificity (95% CI)
			
	Positive (N=17)		Negative (N =118)				
				
	n	%	n	%			
VIA test						70.6 (62.9–78.3)	94.1 (90.1–98.1
Positive	12	70.6	07	5.9	19		
Négative	05	29.4	111	94.1	116		
VIL test						82.4 (75.9 – 88.9)	82.2 (75.7 – 88.7)
Positive	14	82.4	21	17.8	35		
Négative	03	17.6	97	82.2	100		

### Image characteristics for both examiners

Compared to the examiner -1, the examiner -2 found a high percentage of inflammations (red) : 15.3% (n=22) versus 9% (n=13). As for the images of the acidophile, the examiner-2 had a higher percentage, 14.6% (n=21) versus 13.2% (n=19). For a negative iodine reaction, examiner - 2 found a lower percentage than the examiner -1 : 22.2% (n=32) versus 24.4% (n=35).

With regard to smartphonic impressions, the examiner -1 found the normal impressions almost equal to that of the examiner -2, i.e. 72.9% (n=105) versus 72.2% (n=104). (Table 2)

### Smart phonic images

For the two examiners at two sites, the concordance between positive VIA images was 81.0% (n=17/21) and 98.4% (n=121/123) between negative VIA images. The k variability was 0.83. The concordance between positive VIL images was 93.8% (n=30/32) and 95.5% (n=107/112) between negative VIL images, with k variability of 0.86. The concordance between positive smart phonic impressions was 93.8% (n=30/32) and 95.5% (n=107/112) between negative smart phonic impressions, with k = 0.86 (Table 3).

Performance of tests VIA/VIL tests in comparison to cytology at Tchibanga site in 2018

The performance of the VIA and VIL images of the on-site examiner was measured. They were compared to the results of cytology. The sensitivity of the on-site VIA test was 70.6% (95% CI : 62.9 – 78.3), its specificity was 94.1% (95% CI: 90.1 – 98.1) ; the positive predictive value (PPV) was 63.2% and the negative predictive value (NPV) was 95.7%. For the VIL test, the sensitivity was 82.4% (95% CI: 75.9 – 88.9), its specificity 82.2% (95% CI: 75.7 – 88.7); the PPV 40.0% and the NPV 97% (Table 4).

## Discussion

### Limits of study

#### Socio-demographic characteristics

We found the average age of the study participants to be 34 years. The average age we found could be explained on the one hand by the fact that the study population was composed of women aged 18 to 70 and on the other hand by the juvenile nature of the Gabonese population with an average age of 26 years10. Certain authors have worked on the same subject as us6, 11, 12, 13, 14. Among them, there are those who found the average age similar to ours, 34–37 years in Guinea Conakry and Ethiopia6,13, 14. Other studies have found that the age above our age ranges from 39 to 40 years11,12, and that the higher age could be explained by the study population whose extremes ranged from 25 to 65 years.

### Image characteristics for both examiners

The results of our study reveal that the examiner-1 found 16.7% of ATG1 and 7.6% of ATG2 against 16% and 6.2% respectively for the examiner-2.

These results are high for both examiners; the slight difference between them, to the benefit of the examiner -2, could be explained by the level of experience of the two examiners and their training in relation to the screening and diagnosis of precancerous lesions.

The prevalence of dysplasias that we found could be justified by the previous absence of the screening program organized in the province of Nyanga. This result is significantly higher than that of Conakry, which had a 2.6% prevalence of lesions. This high prevalence of precancerous lesions could be justified by the previous absence of an organized screening programme in Nyanga province.

The examiner -1 found a sensitivity, specificity, positive predictive value (PPV) and negative predictive value (NPV) of 70.6%, 94.1%, 63.2% and 95.6% respectively on VIA images. These values were generally better for examiner- 2 (off-site), who found 82.4%, 94.1%, 66.7% and 97.4% respectively. These differences could also be explained by the difference iexperience. The results of examiner -2 (off-site) are similar to those reported by the Brazilian study, which found in 2013 in Brazil a sensitivity of 84.0%, specificity of 95.83% and a higher PPV of 92.78% (k = 0.441) on VIA images14.

Concerning the VIL images, we found that the examiner -1 (on-site) found a sensitivity of 82.4%, a specificity of 82.2%, a PPV of 40% and a NPV of 97%. These values were also better for the examiner -2 (off-site) who found 88.2%, 85.6%, 46.9% and 98.1% respectively. The off-site examiner's results are similar to those of Brazil, for sensitivity at 88.0%, but with higher specificity and PPV at 97.26% and 94.9% respectively (k = 0.533) (14). Other authors had the lowest score in Madagascar in 2015 that showed overall lower values, with a sensitivity of 28.6% (95% CI 3.7–71) and specificity of 87.2% (95% CI 77.7–93.7) for examiners -1 (on site), compared to a sensitivity between 42.9% and 85.7% and specificity between 48.1% and 79.2% for examiners -2(off site)15–16. The result we found states the importance of encouraging training for technicians capable of really diagnosing real cases of dysplasia, in order to promote early care for women, since their contribution to the development of the economy is well established.

### Limitations

This study has some limitations, such as the study population whose minimum age was 18 years instead of 25 years, as recommended by the World Health Organization, that cervical cancer screening should focus on women 25 years of age or older (cervical cancer screening target population). The standard gold in our study was cytology (1) including sensitivity and specificity instead of histology.

## Conclusion

This study found that the average age of participants was 34 years; the examiner-2 had more expertise than the examiner -1. There were a very good concordance between positive smartphonic impressions (93.8%) and (95.5%) between negative smart phonic impressions, with k = 0.86. We also noted that parameters of diagnostic validity were better: sensitivity of the on-site VIA test was 70.6%, specificity was 94.1% ; the positive predictive value was 63.2% and the negative predictive value was 95.7%. Concerning the VIL test, the sensitivity was 82.4%, its specificity 82.2%; the PPV 40.0% and the NPV 97%. The performances are better. This study explored a potential role of telemedicine by using of Smartphone as tool in diagnosis of cervical precancerous lesions in under resourced settings. There is a necessity to use the smartphone as a tool for the diagnosis of precancerous lesions.

## Data Availability

The datasets used and analysed during the current study available from the corresponding author on reasonable request. The datasets generated and/or analysed during the current study are not publicly available due to the promise made to health staff to keep the data confidential when they are questioned but are available from the corresponding author on reasonable request.
